# Phase-Shifted Eccentric Core Fiber Bragg Grating Fabricated by Electric Arc Discharge for Directional Bending Measurement

**DOI:** 10.3390/s18041168

**Published:** 2018-04-11

**Authors:** Yang Ouyang, Jianxia Liu, Xiaofeng Xu, Yujia Zhao, Ai Zhou

**Affiliations:** 1 National Engineering Laboratory for Fiber Optic Sensing Technology, Wuhan University of Technology, Wuhan 430074, China; ouyang_yang123@163.com (Y.O.); XXFCOLDJT@163.com (X.X.); 18603611868@163.com (Y.Z.); 2 School of Information Engineering, Wuhan University of Technology, Wuhan 430074, China; 3 School of Electrical and Information Engineering, Hubei University of Science and Technology, Xianning 437100, China; jxliu2015@126.com

**Keywords:** eccentric core fiber, phase-shifted, electric arc discharge, fiber Bragg grating

## Abstract

A phase-shifted eccentric core fiber Bragg grating (PS-ECFBG) fabricated by electric arc discharge (EAD) is presented and demonstrated. It is composed of a fraction of eccentric core fiber fusion spliced in between two pieces of commercial single mode fibers, where a PS-FBG was written. The EAD in this work could flexibly change the amount of phase-shift by changing the discharge number or discharge duration. Because of the offset location of the eccentric core and the ultra-narrow resonant peak of the PS-ECFBG, it has a higher accuracy for measuring the directional bend. The elongation and compression of the eccentric core keep the magnitude of phase shift still unchanged during the bending process. The bending sensitivities of the PS-ECFBG at two opposite most sensitive directions are 57.4 pm/m^−1^ and −51.5 pm/m^−1^, respectively. Besides, the PS-ECFBG has the potential to be a tunable narrow bandpass filter, which has a wider bi-directional adjustable range because of the bending responses. The strain and temperature sensitivities of the PS-ECFBG are experimentally measured as well, which are 0.70 pm/με and 8.85 pm/°C, respectively.

## 1. Introduction

Phase-shifted fiber Bragg grating (PS-FBG) was firstly introduced by C. M. Ragdale, et al. for obtaining an ultra-narrow-band filter [1]. It is found that more neighboring resonant peaks could be obtained in the spectrum by introducing phase changes in the center of grating [2]. Then, the PS-FBG has received a great deal of attention for its narrow linewidth, multiple channels, and flexible combination, and has been widely used in the fields of optical communication, optical calculation, and optical sensing as an emerging optical passive device [3,4,5,6].

There are many reported techniques for PS-FBG fabrication. A phase-shifted phase mask was used to fabricate a PS-FBG directly [7]. Such a method has good reliability and repeatability, but the location and amount of phase shift cannot be changed flexibly once the expensive phase mask was produced. Fiber gratings that were tuned by resistive Joule heating of a thin metal film deposited onto the fiber were attractive and the location of the phase shift was easy to control, while the influence area and stability of the temperature could not be controlled well [8]. To solve the problem, phase shifts induced by the piezoelectric transducers were presented [9]. This method improved the accuracy, but the exorbitant price of control equipment limited the range of application. A type of PS-FBG based on an in-grating bubble fabricated by femtosecond laser ablation was proposed [10]. Such a method of microstructure has a unique sensing character while it needs a complex fabrication process. Electric arc discharge (EAD) was an important technique for fabricating PS-FBG due to the cheap, simple, and flexible operation, and presented a stable sensing character [11].

Some fiber optic sensors based on PS-FBGs have been developed for sensing various parameters. For instance, the Bragg frequency and the birefringence-introduced frequency of PS-FBG in a polarization maintaining fiber could be measured to perform the discrimination of strain and temperature [12]. The sharp resonance of PS-FBG could be monitored by a continuous-wave laser to measure ultrasound-induced pressure variations [13]. An extrinsic PS-FBG by separating two identical FBG reflectors with a micrometer air gap as a phase-shift was presented to be a compact refractometer [14]. The refractive index of the liquid filled in the gap would influence the transmission spectrum. Those sensors mentioned above suggested that the PS-FBGs have a variety of sensing information to be utilized. As for bending measurement, there are many kinds of structures, such as Mach–Zehnder interferometers (MZIs), long period fiber gratings (LPFGs), FBGs, and so on [15,16,17,18,19]. The MZI-based sensor has a highly bending sensitivity, but the interference signal has multiple resonance peaks whose sensitivities are quite different. The LPFG-based sensor has good reproducibility, while the measurement range of curvature is small in general. The FBG-based sensor for bending has a good linear correlation with large measurement range, while the resolution of sensing detection is lower than that of the PS-FBG. 

In this paper, a PS-FBG in eccentric core fiber (ECF) fabricated by EAD method for directional bending measurement is presented. The EAD in this work could flexibly change the phase-shift of the PS-FBG by changing the number or time of discharges. Compared with the ordinary ECF-based FBG, the proposed PS-FBG in ECF has a higher accuracy for sensing detection because of the ultra-narrow resonant peak. Compared with the ordinary PS-FBG written in single mode fiber (SMF), the proposed PS-ECFBG is sensitive to directional bending and has the potential to be a tunable narrow bandpass filter with a wider bi-directional adjustable range.

## 2. Sensor Fabrication and Working Principle

**Figure 1:**
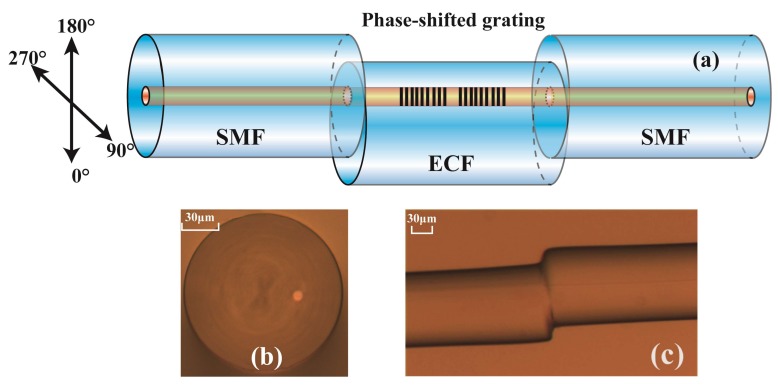
(**a**) Schematic diagram of the PS-ECFBG. (**b**) Microscope image of the ECF, and (**c**) splice junction between the ECF and the SMF.

The schematic diagram of the PS-ECFBG is shown in Figure 1a. It consists of a fraction of ECF fusion spliced in between two pieces of commercial SMFs, where a PS-FBG fabricated by EAD was written. The microscope image of the ECF is shown in Figure 1b. It is composed of a core located ~27 μm away from the central axis of the ECF and a conventional cladding, whose diameters are about 8.5 μm and 125 μm, respectively. The core and cladding diameters of the commercial SMF in this work are ~8.5 μm and 125 μm, respectively.

**Figure 2:**
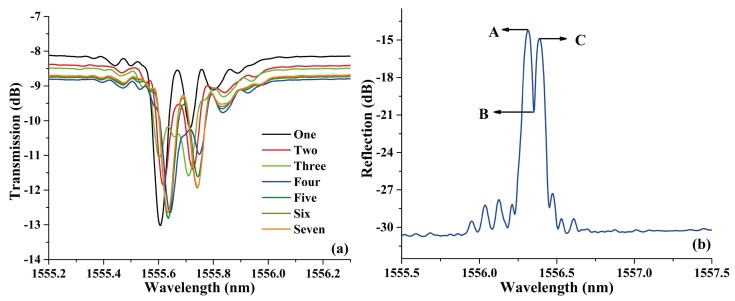
(**a**) The evolutions of the transmission spectrum of the PS-FBG written in the ECF during discharge process, and (**b**) the reflection spectra of the PS-ECFBG by seven discharges.

The fabrication process is illustrated in detail as follows, firstly, the ECF was hydrogen-loaded in a hydrogen chamber for 12 days at high pressure (10Mpa) and room temperature (26 °C) to enhance the photosensitivity [20]. Secondly, a fraction of ECF, with a length of ~3 cm, was fusion spliced in between two SMFs using a commercial fiber fusion splicer (Fujikura FSM-60S) under the manual model. The arc discharge current and time were standard minus 11.5 mA and 1100 ms, respectively. The splice junction between the ECF and the SMF is shown in Figure 1c. Thirdly, a FBG was written in the ECF by using a 248 nm KrF excimer laser and a phase mask plate with a length of 2 cm. Then, the ECF-based FBG was cut into two sections at the middle of the grating using a fiber cleaver. At last, we used a polarization maintaining fiber fusion splicer (Fujikura FSM-100P) to rotate the two sections of the proposed ECF and fusion spliced them again. It is worth noting that both the cleaving and rotating step can be omitted if we add a stress monitoring device on the fusion splicer, which reduces the complexity and improves the precision effectively. At the same time, the fusion splicer here was used to realize several arc discharge at the splice junction between the two sections of ECFs, and the forming process of the PS-FBG versus discharge times is shown in Figure 2a. The arc discharge current and time are 12.4 mA and 3000 ms, respectively. From the figure, the phase shift of the PS-FBG varied with the number of discharges, which means that PS-FBGs with different phase shift could be fabricated by only changing the discharge number or discharge duration. The reflection spectrum of the PS-FBG made by seven discharges is shown in Figure 2b. The 3 dB bandwidth of the ECF-FBG is 0.145 nm, and the bandwidth of the transmission window is about 0.025 nm. The insertion loss caused by one discharge is about 0.3 dB. The central wavelength is 1556.35 nm.

**Figure 3:**
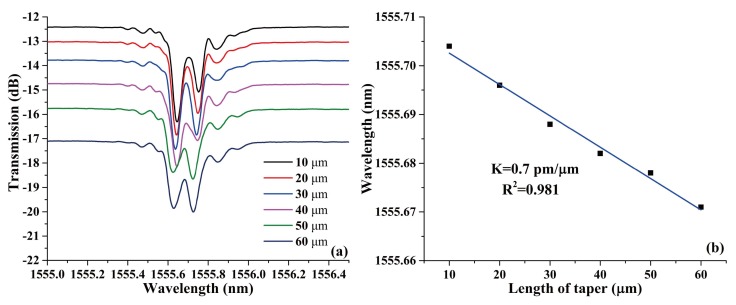
(**a**) The evolution of transmission spectrum versus the length of taper, and (**b**) the corresponding linear fit of the transmission window in PS-ECFBG.

The function of the EAD technique in this work is to erase the effective modulation of FBG. Because there is no definite linear relationship between the length of the erased FBG and the number of discharges, fusion tapering method based on EAD was employed to quantitatively erase the FBG and fabricate a set of PS-FBGs with determinate phase shifts. The evolution of the transmission spectrum versus the length of the taper and the corresponding linear relation are shown in Figure 3a,b, respectively. From the figures, it can be seen that the location of the transmission windows in the PSFBG changes linearly with the increase of the taper length. This phenomenon could also be explained by the changes in length of the fused taper altering the magnitude of phase shift. Besides, because of the offset location of the eccentric core, the light is easier to leak from the core to the cladding when the ECF is deformed. Thus, the relatively large loss comes mainly from the tapering, compared with that of the SMF.

As for bending measurement, the wavelength of PS-FBG will shift because of the eccentric core of the ECF. The wavelength of the FBG could be obtained by the equation

(1) $$\lambda_{B}=2n_{\mathrm{eff}}\Lambda$$

where $n_{\mathrm{eff}}$ is the effective refraction index of a fiber and Λ is the grating period. The wavelength shift versus curvature is

(2) $$\frac{{d\lambda }_{B}}{\mathrm{dC}}=2n_{\mathrm{eff}}\frac{d\Lambda }{\mathrm{dC}}+2\Lambda \frac{{\mathrm{dn}}_{\mathrm{eff}}}{\mathrm{dC}}$$

The phase shift in the center of ECF is

(3) $$\varphi =\frac{2{\pi n}_{\mathrm{eff}}L}{\lambda_{B}}$$

where L is the equivalent length of discharge erased length. Therefore, the variation of the φ versus curvature can be written as

(4) $$\frac{d\varphi }{\mathrm{dC}}=\frac{2{\pi n}_{\mathrm{eff}}}{\lambda_{B}}\frac{\mathrm{dL}}{\mathrm{dC}}+\frac{2\pi L}{\lambda_{B}}\frac{{\mathrm{dn}}_{\mathrm{eff}}}{\mathrm{dC}}-\frac{2{\pi n}_{\mathrm{eff}}L}{\lambda_{B}{}^{2}}\frac{{d\lambda }_{B}}{\mathrm{dC}}$$

Due to the same material of the fiber, the following equation is true

(5) $$\frac{\mathrm{dl}}{\mathrm{dC}}=\frac{L}{\Lambda }\frac{d\Lambda }{\mathrm{dC}}$$

Substituting Equation (1), (2), and (5) into Equation (4), we can get

(6) $$\frac{d\varphi }{\mathrm{dC}}=0$$

Therefore, the wavelength of the PS-ECFBG will shift with bending while the magnitude of the phase shift keeps unchanged during the bending process. The same is true for strain and temperature.

## 3. Results and Discussion

**Figure 4:**
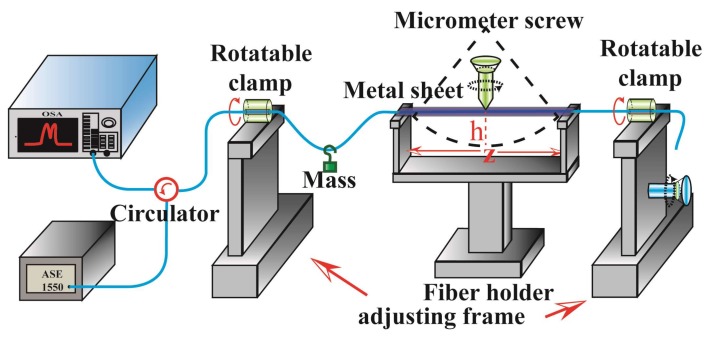
Schematic diagram of the experimental setup for directional bending and strain measurement.

The experimental setup for measuring directional bend and strain is shown in Figure 4. The PS-FBG written in the ECF was placed at the middle of the fiber holder with a metal sheet covered. A tunable curvature from 0 m^−1^ to 7.55 m^−1^ could be realized by screwing the micrometer screw. The specific figure of the curvature is calculated by the formula *C* = 2*h*/[*h*^2^ + (2*z*)^2^], where z and h are the length of the fiber holder and bending displacement of the sensing structure, respectively. The two rotatable clamps with a division value of 5° are used to rotate the sensing element realizing the directional bending measurement from 0° to 360°. A mass with a weight of 3 g is hung on the SMF for keeping the PS-FBG straight with nearly no strain induced during the bending process. The detailed process to identify the directional angles of the ECF is as follows: firstly, the sensing element is put at the middle of the fiber holder with a metal sheet covered. Secondly, the microscope is used to identify the directional angles of the ECF roughly. Thirdly, the numerical value of the wavelength shift is recorded when bending the sensor at the curvature of 7 m^−1^. Fourthly, the two clamps are rotated 5° and the third step is repeated. Therefore, the exact direction of 0° can be identified until the largest red-shift of wavelength occurs. Additionally, two adjusting frames are used to change the length of the fiber for measuring the strain response, with a division value of 10 μm. The light emitted by a 1550 nm amplified spontaneous emission (ASE) is detected by an optical spectrum analyzer (OSA) through a circulator and the PS-ECFBG, respectively.

**Figure 5:**
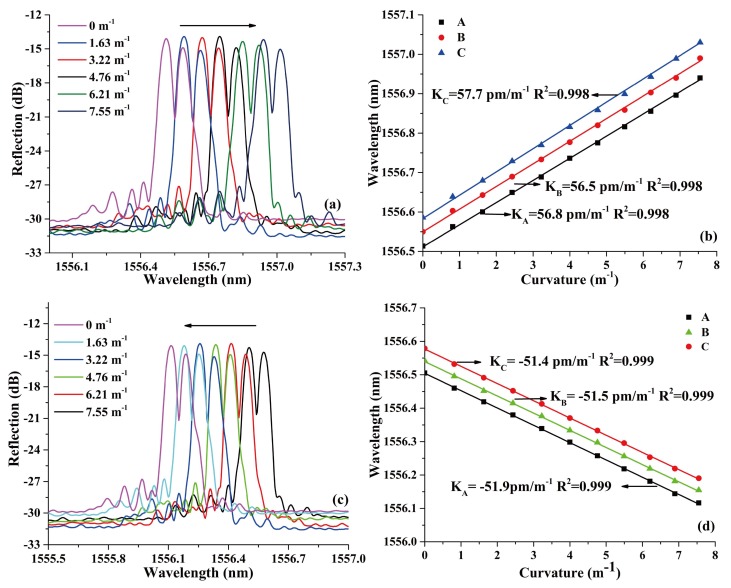
Spectral responses of the PS-ECFBG under different curvatures at (**a**) 0° and (**c**) 180°, and (**b**,**d**) the corresponding linear fit of peaks A, B, and C.

The reflected spectra variation of the PS-ECFBG at bending direction of 0° and 180° are shown in Figure 5a,c, respectively. The wavelength shift versus curvature of the three feature points—A, B, and C—as presented in Figure 2b are shown in Figure 5b,d, respectively. For the PS-ECFBG, all the resonant peaks have a red shift and blue shift with the increase of the curvature at the bending direction of 0° and 180°, respectively. This is due to the elongation and compression of the eccentric core of the ECF. At the same time, the magnitudes phase-shifts change slightly with the variation of curvature, which does not agree well with the result of the previous theoretical analysis. That is because the two rotatable clamps cannot guarantee an identical rotation angle, resulting in a slight torsion of the sensing element, and the curvature in the metal sheet cannot guarantee a complete uniformity. These two errors in our experimental setup made a different changes between the region of Bragg grating and the discharge erased region. Therefore, the magnitudes in peaks A, B, and C have a variation. The bending sensitivities of A, B, and C are almost the same at bending direction of 0° and 180°, which are 56.2, 57.4, 59.0, −51.9, −51.5, and −51.4 pm/m^−1^, respectively. At 90° and 270°, the reflected spectrum remains unchanged because the eccentric core is in the neutral plane of the bending. Besides, the PS-ECFBG is formed by introducing a phase shift in a uniform ECFBG, which opens an ultra-narrow transmission window on the reflected spectrum. The bandwidth of the ECF-FBG is 0.145 nm, and that of the transmission window is about 0.025 nm. Therefore, compared with the ECFBG, it has a better wavelength selectivity and peak searching.

**Figure 6:**
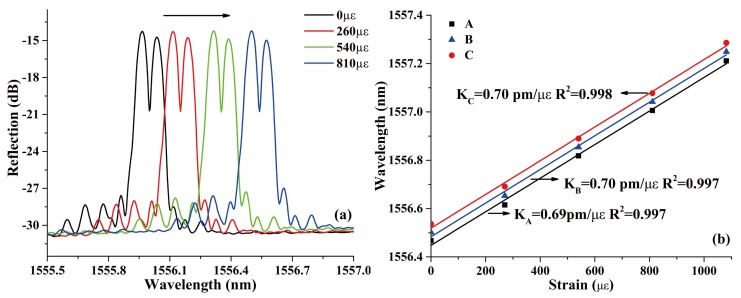
(**a**) Spectral responses of the PS-ECFBG under different strain and (**b**) the corresponding linear fit of peaks A, B, and C.

For strain responses, the evolutions of the reflection spectrum and the corresponding linear fit of peaks A, B, and C are shown in Figure 6a,b, respectively. The resonant peaks shift toward longer wavelength with the increase of the applied strain from 0 με to 1100 με, which is mainly caused by the elasto-optical effect of silica fiber. The strain sensitivities of A, B, and C are 0.69, 0.70, and 0.70 pm/με, respectively. The almost same sensitivities shows that the increased axial strain did not change the relative phase shift of the PS-ECFBG. It has to be noticed that the sensitivity is smaller than that of the ordinary PS-FBG in SMF (~1 pm/με) [11], because of the smaller contact area of the ECF-SMF joint compared with the cross-section area of the ECF and the SMF.

**Figure 7:**
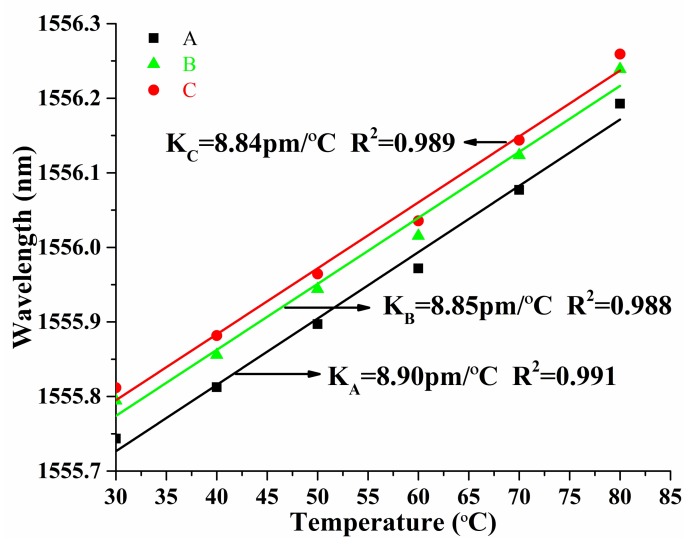
Linear relationship between the wavelength of extreme point of the reflection spectra and the temperature.

The temperature response characteristics of the PS-ECFBG were also investigated, and Figure 7 shows the experimental results. All the wavelengths of A, B, and C have a red shift with the increase of temperature from 30 °C to 80 °C. This is mainly due to the thermo-optic effect and the thermal expansion effect of silica fiber. The temperature sensitivities of A, B, and C are 8.90, 8.85, and 8.84 pm/°C, respectively. The temperature sensitivity of an ordinary PS-FBG in SMF is about 10 pm/°C [11]. Such differences could be explained as follows. The temperature sensitivity of PS-FBG is mainly caused by the thermal optical coefficient, which is determined by the doping concentration of the fiber, and the ECF is a home-made fiber whose doping concentration is lower than that of the commercial SMF. Thus, the sensitivity is smaller than that of the SMF.

## 4. Conclusions

In conclusion, a PS-ECFBG fabricated by EAD was presented and demonstrated. The EAD here was used to form a tunable phase shift. Because of the offset location of the eccentric core in ECF, the proposed structure is sensitive to directional bend and could be used as a high-accuracy bending sensor. In addition, the elongation and compression of the eccentric core during bending process did not change the relative phase-shift magnitude. Therefore, the bending sensitivities of extreme point of the reflection spectra are respectively 56.2, 57.4, and 59.0 pm/m^−1^ at a bending direction of 0° and −51.9, −51.5, and −51.4 pm/m^−1^ at a bending direction of 180°. It could also be used as a tunable narrow bandpass filter, which has a wider bi-directional adjustable range because of the bending responses. We also measured the strain and temperature sensitivities of extreme point of the reflection spectra, which are 0.69, 0.70, and 0.70 pm/με; and 8.90, 8.85, and 8.84 pm/°C, respectively.
